# Author Correction: Probabilistic embedding, clustering, and alignment for integrating spatial transcriptomics data with PRECAST

**DOI:** 10.1038/s41467-023-42412-1

**Published:** 2023-10-18

**Authors:** Wei Liu, Xu Liao, Ziye Luo, Yi Yang, Mai Chan Lau, Yuling Jiao, Xingjie Shi, Weiwei Zhai, Hongkai Ji, Joe Yeong, Jin Liu

**Affiliations:** 1 Centre for Quantitative Medicine, Health Services & Systems Research, Duke-NUS Medical School, Singapore, Singapore; 2 School of Statistics, Renmin University, Beijing, China; 3 Institute of Molecular and Cell Biology (IMCB), Agency of Science, Technology and Research (A*STAR), Singapore, Singapore; 4 School of Mathematics and Statistics, Wuhan University, Wuhan, China; 5 Academy of Statistics and Interdisciplinary Sciences, East China Normal University, Shanghai, China; 6 Key Laboratory of Zoological Systematics and Evolution, Institute of Zoology, Chinese Academy of Sciences, Beijing, China; 7 Department of Biostatistics, Johns Hopkins Bloomberg School of Public Health, Baltimore, MD USA; 8 Department of Anatomical Pathology, Singapore General Hospital, Singapore, Singapore; 9 School of Data Science, The Chinese University of Hong Kong-Shenzhen, Shenzhen, China

**Keywords:** Data integration, Statistical methods, Software, Bioinformatics

Correction to: *Nature Communications* 10.1038/s41467-023-35947-w, published online 18 January 2023

In this article, the funding from the ‘University Development Fund (UDF01003033) from The Chinese University of Hong Kong, Shenzhen’ was omitted. The original article has been corrected.

