# Supplementary material for: Probabilistic embedding, clustering, and alignment for integrating spatial transcriptomics data with PRECAST
Source: Nat Commun. 2023 Jan 18;14:296. doi: 10.1038/s41467-023-35947-w (PMC9849443; doi:10.1038/s41467-023-35947-w)
Supplement: Supplementary file 3 — Description of Additional Supplementary Files [file 41467_2023_35947_MOESM3_ESM.pdf]

Descriptions of additional supplementary files

Supplementary Data 1: The domain-specific differentially expressed (DE) genes for each domain identified by PRECAST for the 12 dorsolateral prefrontal cortex Visium sections. The genes were identified as DE genes with adjusted p-values of less than 0.001 and a log-fold change of greater than 0.25. The p-values are based on a Wilcoxon Rank Sum test.

Supplementary Data 2: The significant gene list associated with the estimated pseudotime with adjusted p-values of less than 0.001 for the 12 dorsolateral prefrontal cortex Visium sections. The p-values are based on tow-sided Pearson's product moment correlation coefficient test.

Supplementary Data 3: The identified spatially variable genes (SVGs) of each dorsolateral prefrontal cortex Visium section in SVA analysis based on the aligned embeddings from PRECAST. The genes were identified as SVGs at a false discovery rate (FDR) of 1%. The p-values are calculated and calibrated by SPARK.

Supplementary Data 4: The domain-specific DE genes for each domain identified by PRECAST for the eight mouse liver sections. The genes were identified as DE genes with adjusted p-values of less than 0.001 and a log-fold change of greater than 0.25. The p-values are based on a Wilcoxon Rank Sum test.

Supplementary Data 5: The domain-specific DE genes for each domain identified by PRECAST for the 16 mouse olfactory bulb sections with reduced resolution. The genes were identified as DE genes with adjusted p-values of less than 0.001 and a log-fold change of greater than 0.25. The p-values are based on a Wilcoxon Rank Sum test.

Supplementary Data 6: The domain-specific DE genes for each domain identified by PRECAST for the four human HCC Visium sections. The genes were identified as DE genes with adjusted p-values of less than 0.001 and a log-fold change of greater than 0.25. The p-values are based on a Wilcoxon Rank Sum test.

Supplementary Data 7: The identified spatially variable genes (SVGs) of each human HCC Visium section in SVA analysis based on the aligned embeddings from PRECAST. The genes were identified as SVGs at a FDR of 1%. The p-values are calculated and calibrated by SPARK.

Supplementary Data 8: Simulation settings of mean and covariance components in the Gaussian mixture model of simulation scenarios 1 to 5.
